# Deep learning strategies with CReToNeXt-YOLOv5 for advanced pig face emotion detection

**DOI:** 10.1038/s41598-024-51755-8

**Published:** 2024-01-19

**Authors:** Lili Nie, Bugao Li, Yihan Du, Fan Jiao, Xinyue Song, Zhenyu Liu

**Affiliations:** 1 College of Information Science and Engineering, Shanxi Agricultural University, Taigu, 030801 Shanxi China; 2 College of Animal Science, Shanxi Agricultural University, Taigu, 030801 Shanxi China; 3 College of Agricultural Engineering, Shanxi Agricultural University, Taigu, 030801 Shanxi China

**Keywords:** Computational biology and bioinformatics, Zoology

## Abstract

This study underscores the paramount importance of facial expressions in pigs, serving as a sophisticated mode of communication to gauge their emotions, physical well-being, and intentions. Given the inherent challenges in deciphering such expressions due to pigs' rudimentary facial muscle structure, we introduced an avant-garde pig facial expression recognition model named CReToNeXt-YOLOv5. The proposed model encompasses several refinements tailored for heightened accuracy and adeptness in detection. Primarily, the transition from the CIOU to the EIOU loss function optimized the training dynamics, leading to precision-driven regression outcomes. Furthermore, the incorporation of the Coordinate Attention mechanism accentuated the model's sensitivity to intricate expression features. A significant innovation was the integration of the CReToNeXt module, fortifying the model's prowess in discerning nuanced expressions. Efficacy trials revealed that CReToNeXt-YOLOv5 clinched a mean average precision (mAP) of 89.4%, marking a substantial enhancement by 6.7% relative to the foundational YOLOv5. Crucially, this advancement holds profound implications for animal welfare monitoring and research, as our findings underscore the model's capacity to revolutionize the accuracy of pig facial expression recognition, paving the way for more humane and informed livestock management practices.

## Introduction

With growing concerns surrounding animal welfare in legal and social spheres, the application of machine vision in ensuring animal well-being has seen a remarkable upsurge. This technology, by facilitating precise livestock condition detection, not only simplifies management practices but also significantly curtails costs. Noteworthy applications include the pioneering work by Meiqing et al.^1^ where re-ID features were harmoniously integrated with intersection over the union to adeptly detect and re-identify pigs, providing invaluable insights into their health status. The melding of Gabor and LBP features with the Support Vector Machine (SVM) has further accelerated automated pig behavior analyses^2^. Thus, the intersection of machine learning and animal welfare offers fertile grounds for transformative research.

The intricate world of animal emotions has come to the forefront in contemporary animal welfare research^3^. Expressions in animals, intricate tapestries woven from physiological, psychological, and behavioral threads, act as vital emotional barometers^4^. Highlighting the depth of this realm, it's noteworthy that chimpanzees possess as many as 23 facial muscle groups, emphasizing the pivotal role of facial muscles in emotional communication^5^. Pigs, in their own unique way, convey emotional cues through ear positions^6^, and their facial expressions can swing between signs of aggression to those of fear, providing foundational knowledge for their facial expression categorization^7^.

While the domain of human facial recognition has witnessed paradigm shifts due to machine vision, its application to animals, particularly pigs, has similarly shown promising advancements. A testament to this is the staggering 96.7% accuracy achieved using three facial recognition methods on pigs in natural settings^8^. Prioritizing animal welfare, convolutional neural networks were developed to distinguish pig stress levels, integrating Grad-CAM to pinpoint areas demanding attention^9^. The fusion of Haar features with shallow convolutional neural networks yielded an 83% accuracy in pig face recognition^10^. Further innovations, like combining the ResNAM network with the SphereFace loss function, propelled the recognition rate to 95.28%^11^. The cascaded LSTM framework, endowed with multiple attention mechanisms, has shown prowess in discerning facial expressions, boasting an average accuracy of 91.82% for four distinct expressions ^12^.

Given the expansive growth in machine vision applications, advancements in cow identification and management have also come into focus. Pioneering works such as those by Kawagoe et al. demonstrated the potential of facial region analysis for distinct cow identification and the estimation of feeding times, showcasing its utility in the early diagnosis of diseases and movement disorders in cows^13^. Furthermore, Zin and her team have highlighted the sophistication of integrating ear tag visual analysis, evolving the tracking systems in the agricultural domain. Validated under actual farm conditions, their methodologies achieved high accuracy in head detection and ear tag recognition, paving the way for intelligent, real-time farm management^14,15^. In another significant contribution, Phyo and Zin introduced a hybrid approach that leverages rolling skew histograms and neural network techniques. Their system effectively identifies cows in dairy rotary parlors, presenting a cost-effective alternative that's non-intrusive to the cows^16^. These advancements in cow-focused research complement the progress observed in pig recognition, emphasizing the profound implications of machine vision in the contemporary realm of animal welfare and management.

The landscape of pig face recognition has undoubtedly evolved, yet there remains a glaring gap in methodologies aimed at understanding their expressions. Given the simplistic facial muscle configuration in pigs and the complexities intrinsic to expression detection, this is a monumental challenge. Addressing this, we introduced the CReToNeXt-YOLOv5-based pig expression recognition model. This model's vision extends beyond mere identification; it seeks to proactively gauge the emotional nuances of pigs, paving the way for prompt interventions and subsequently reducing potential adversities. Ethically, recognizing and responding to pig emotions can profoundly elevate animal welfare standards, presenting both a moral and practical obligation. As such, our primary research objective is to optimize and validate the efficacy of the CReToNeXt-YOLOv5 model in real-world scenarios. The inception of this model not only holds vast transformative promise but also signifies a progressive stride in the realm of animal emotional studies.

## Methods

### Experimental data

#### Pig facial expression collection

**Figure 1:**
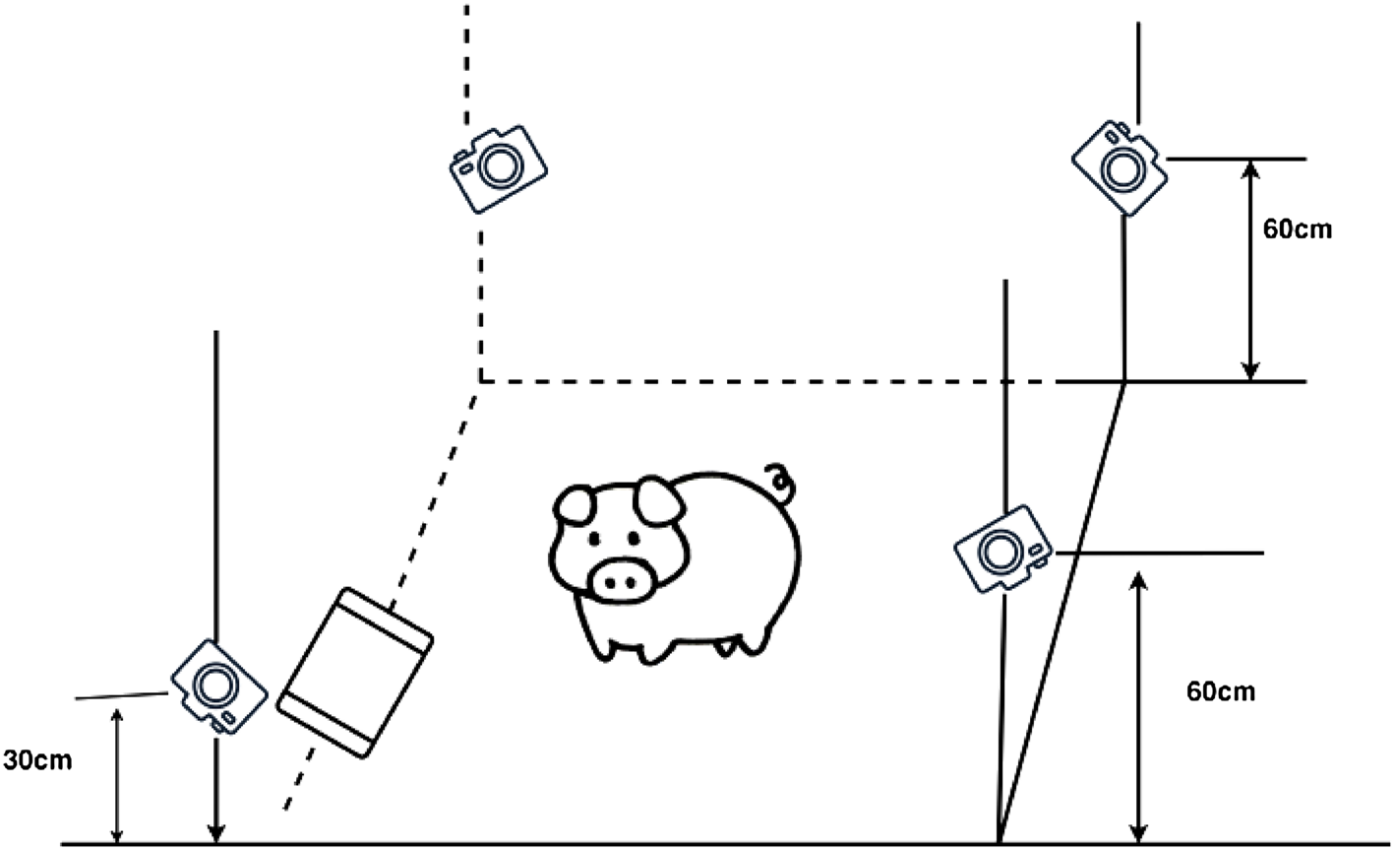
Diagram of the video capture solution.

The database used in this study was collected from pig farms in Shanxi Province. The farms were divided into two areas, each measuring 5 m × 3 m, resulting in a total area of 30m^2^. A total of 20 Landrace pigs, aged 5 months and with an average weight of 68 kg, were selected and reared in captivity for data collection. The data collection period spanned from April to June 2022, between the hours of 07:00 and 16:00. Crucially, capturing genuine and spontaneous pig expressions required a carefully planned camera setup. Three cameras, positioned at an elevated height based on the average vertical size of the pigs, covered the majority of the enclosure. Meanwhile, a fourth camera, strategically placed by the trough, focused on feeding behaviors. This setup, as detailed in Fig. 1, utilized a Canon EOS 700D camera fitted with an EF-S 18–55 mm 1:3.5–5.6 STM lens to produce high-resolution video clips, capturing both frontal and lateral pig facial expressions. Different expression datasets were obtained for scenes with varying states, and both frontal and lateral facial expressions were recorded. A total of 40 video clips were captured, each with a duration of 3–5 h at a frame rate of 25 fps. The images were manually extracted and categorized, and then they were annotated by observing them in the actual video scenarios (e.g., videos of pigs in a cheerful mood while eating and videos of pigs displaying anger during a struggle).

**Figure 2:**
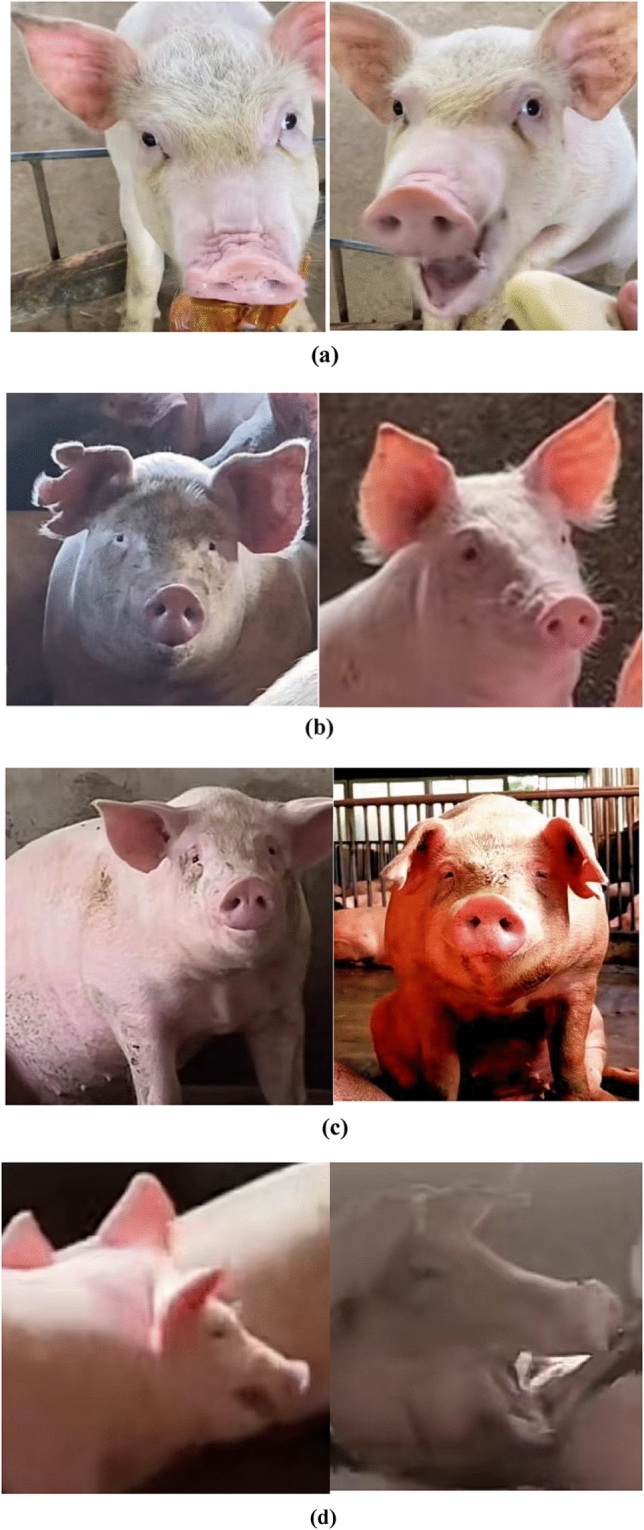
Diagram of the four categories: (**a**) for happy, (**b**) for neutral, (**c**) for fear, (**d**) for anger.

However, the inherent challenges of such a dynamic environment, including varied pig movements and potential inconsistencies between cameras, necessitated stringent data validation. Any imagery affected by rapid pig movements, unanticipated obstructions, or unfavorable lighting was deemed "invalid" and subsequently discarded. As illustrated in Fig. 2, this rigorous filtering process resulted in a diverse yet reliable dataset of 2700 categorized images.

#### Pig facial expressions labelled

Expression labeling is based on relevant research findings^7,12^. For example, during aggressive behavior, pigs exhibit deepened nose wrinkles. When retreating from a failed struggle, the pig's ears droop and its eyes partially open. Startled animals tend to close their eyes and flatten their ears to reduce sensory exposure. The results reveal that facial expressions convey information about aggression in successful struggles and emotional states of fear. The expressions are categorized as follows: "happy," when the pig slightly closes its eyes, raises its upper lip, exposes its canine teeth, and pulls its ears back, usually observed during feeding; "angry," when the pig raises its upper lip, tightens its canine muscles, deepens nose wrinkles, and widens its eyes, typically during an attack; "fear," when the pig slightly closes its eyes, lowers its ears, and reduces nose wrinkles, usually seen in a frightened state or when the defeated side retreats after a struggle; "neutral," when the pig is in a more peaceful state with no significant changes in its facial features. Changes in pig expressions primarily manifest in the eyes, nose, lips, and ears. Table 1 provides descriptions of the facial expression categories and the corresponding scenarios. The expression dataset was created by annotating the pig's facial images in conjunction with the situational context and regional representations observed by pig breeding experts and breeders in the video.

**Table 1:** Description of facial expression categories.

Category	Facial changes				Scene context
	Eye	Nose	Lip	Ear	
Happy	Eyes slightly closed	None	Pull up the upper lip	Pull back the ears	Generally in the eating state
Neutral	None	None	None	None	Normalcy
Fear	Eyes slightly closed	Reduced wrinkles	None	Droopy ears	Generally in a state of shock
Anger	Eyes wide open	Deepened wrinkles	Pull up the upper lip	None	Generally before initiating an act of aggression

**Figure 3:**
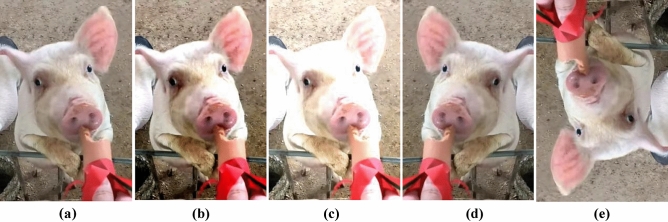
Pig expression image data expansion.

Pig facial expression dataset expansion Before the dataset expansion, the initial set of images underwent a series of pre-processing steps to ensure consistency and enhance the quality for training. All images were resized to a standard resolution, ensuring uniformity across the dataset. Furthermore, any noise reduction or normalization techniques were applied, preparing the images for the subsequent augmentation steps. For data augmentation, geometric transformation methods such as rotation, flipping, cropping, etc., and pixel transformation methods such as adjusting HSV contrast, brightness, saturation, etc., are employed. By applying operations such as contrast enhancement (Fig. 3b), brightness enhancement (Fig. 3c), image flipping (Fig. 3d), and angle rotation (Fig. 3e) to the training images (Fig. 3a), a network with stronger generalization ability can be obtained. This helps reduce overfitting and expands the dataset size. Post augmentation, the dataset size surged to 5400 images. To facilitate effective pig expression recognition, labeling software was employed to annotate the facial regions of pigs within video frame images. This annotation process was meticulous, ensuring that each expression was correctly labeled based on the facial features and the situational context. Subsequently, out of the total images, 3780 were earmarked for training, 1080 for validation, and the remaining 540 for testing.

#### Training environment configuration

The hardware environment is configured with a 12vCPU Intel(R) Xeon(R) Platinum 8255C CPU @ 2.50 GHz and an RTX 3090 GPU with 24 GB of memory. The software environment is supported by Cuda 11.3, and the deep learning framework used is PyTorch 1.11.0. The programming language is Python 3.8, and the toolkit includes PyTorch, OpenCV, and sklearn. The number of training iterations is set to 300, and the batch size is set to 8.

### YOLOv5s model

**Figure 4:**
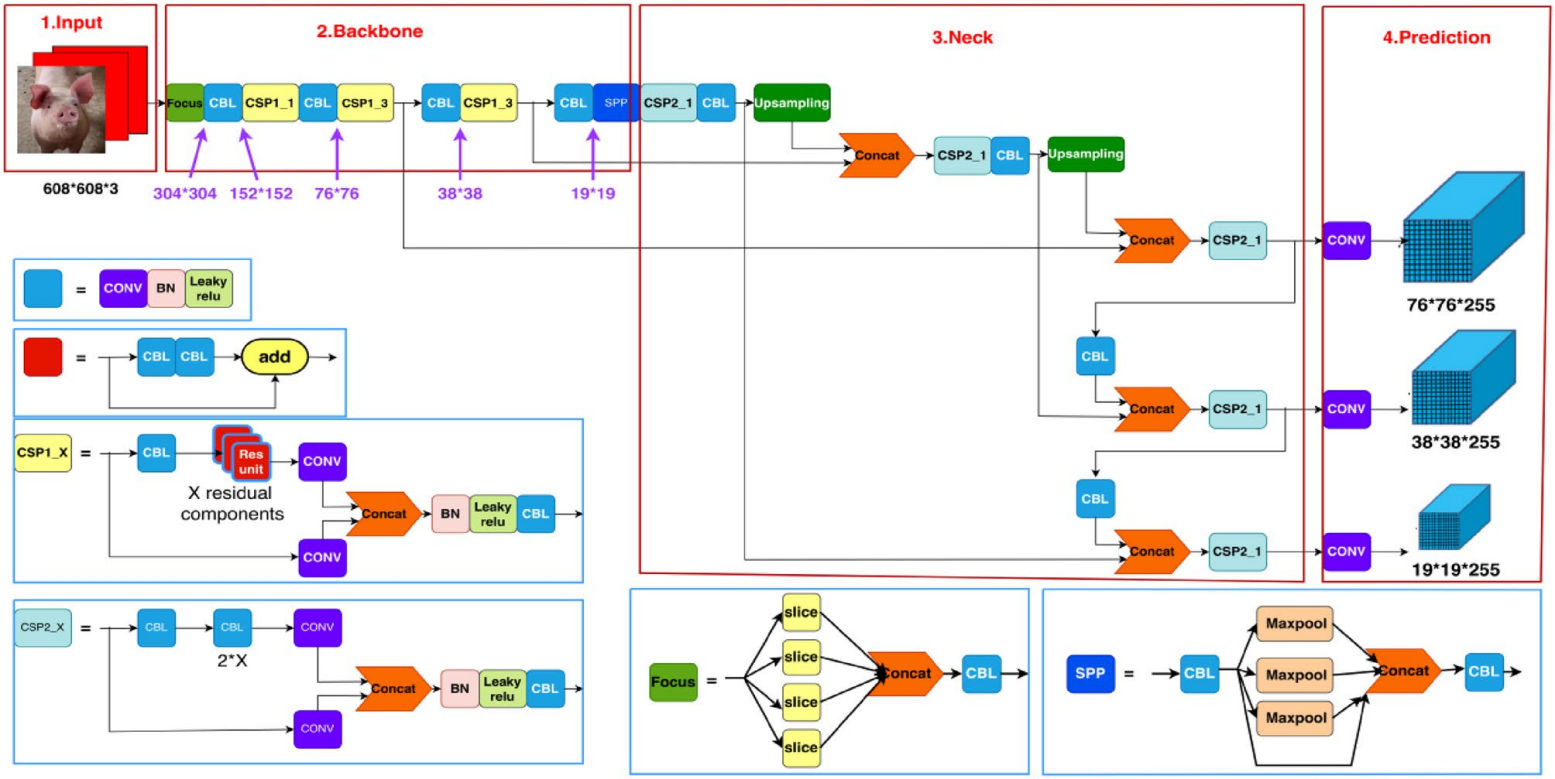
YOLOv5s network structure: input, backbone, neck, and head (prediction).

YOLOv5 can be categorized into four models based on its network depth and breadth, namely s, m, l, and x. In order to improve recognition speed, the YOLOv5s model with the smallest network depth and width was chosen as the base model for pig facial expression recognition. The network structure consists of four parts^17^: Input, Backbone, Neck, and Head (Prediction), as illustrated in Fig. 4.

#### Input

The concept of Mosaic data augmentation involves randomly selecting four images and applying random cropping, flipping, scaling, and other transformations to them. These transformed images are then merged into a single image in a randomly distributed manner. This technique significantly enhances the dataset and increases the batch_size, thereby improving training efficiency.

The innovation of YOLOv5 lies in the incorporation of anchor box calculation during training. During the training process, the anchor box size is dynamically adjusted by predicting a bounding box based on the initial anchor box set, comparing it with the ground truth, calculating the loss, and updating the anchor box size accordingly. This approach allows YOLOv5 to adapt and calculate the optimal anchor box values for different training datasets.

The images used in the YOLO algorithm are typically resized to a scale of 1:1. However, in real-world detection projects, the aspect ratio of images varies. Forcing padding to maintain a 1:1 ratio results in additional black edges, which introduce redundant information and impact the inference speed. YOLOv5 has been optimized to address this issue by following these steps: calculating the scaling ratio, obtaining different scaling ratios for the length and width of the image, selecting the smaller scaling factor, and multiplying the length and width by that factor. To ensure that both sides are multiples of 32, padding is applied to the shorter sides. This approach significantly reduces the number of black edges introduced after scaling, thereby reducing the computational load for inference and improving the speed of target detection.

#### Backbone

**Figure 5:**
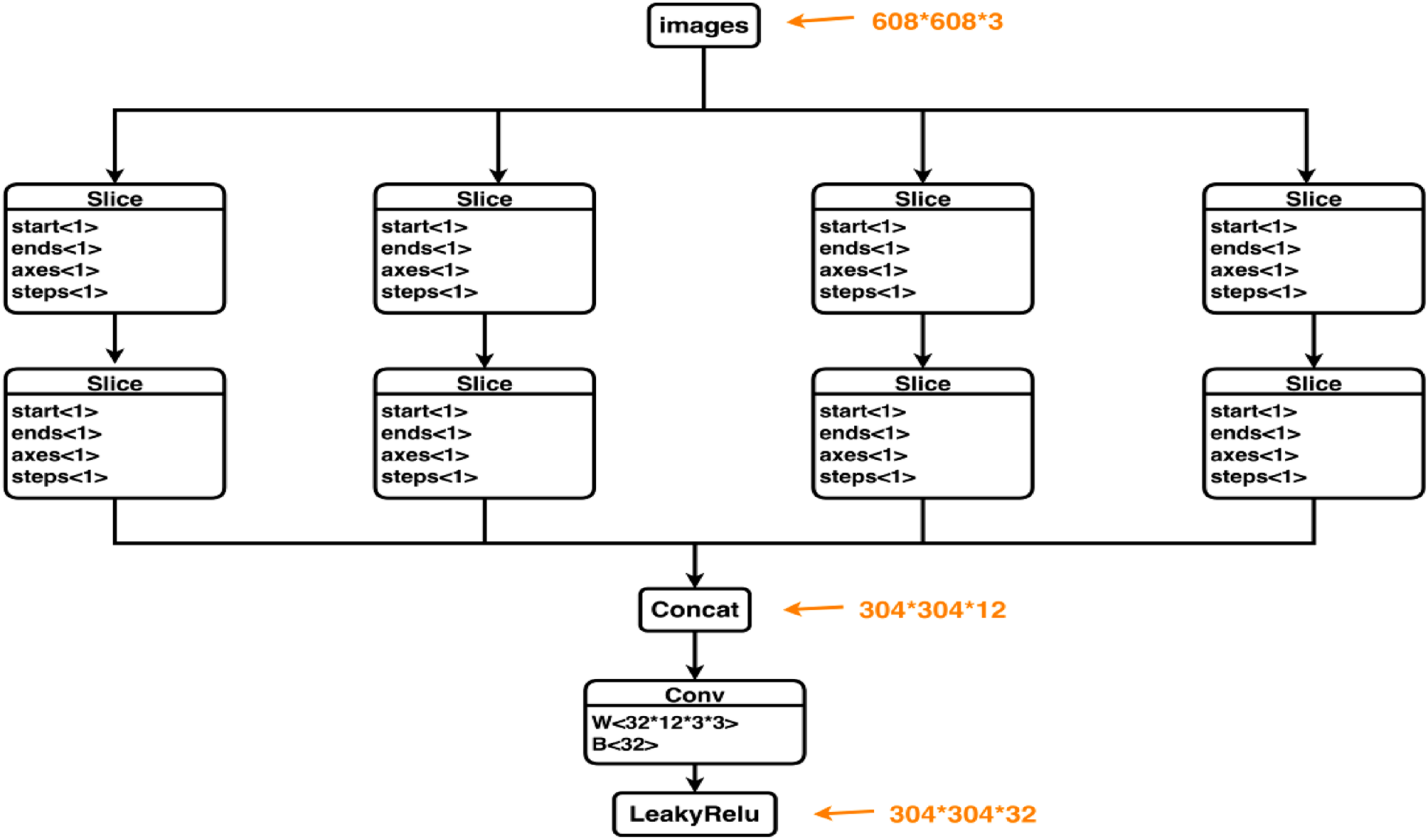
Focus structure.

The backbone network plays a crucial role in extracting information and features from the input images. In Fig. 5, assuming the input image is 608 × 608 × 3, it undergoes a downsampling operation to generate a 304 × 304 × 12 feature map. A convolutional kernel is then applied at the end of the Focus module to convert it into a 304 × 304 × 32 feature map. This cut structure helps to preserve more detailed and fine-grained features.

#### Neck

The Neck module plays a crucial role in effectively integrating the features extracted by the backbone network and enhancing the overall performance of the network. By combining the top-down and bottom-up processes, the Neck module enhances the network's ability to incorporate both semantic and localization information, resulting in improved performance and accuracy.

#### Head (prediction)

The Head part is mainly used to detect the target, and Head expands the number of channels by 1 × 1 convolution for each of the different scales of feature maps obtained in Neck, and the number of expanded feature channels is:

1 $${\text{C}}=({{\text{N}}}_{{\text{c}}}+5)\times {{\text{N}}}_{{\text{a}}}$$

${{\text{N}}}_{{\text{c}}}$ denotes the number of categories; ${{\text{N}}}_{{\text{a}}}$ represents the number of anchors on the respective detection layer. Each value in the '5' represents the horizontal and vertical coordinates, width, height, and confidence level of the center point in the prediction box. The confidence level indicates the certainty of the prediction and is expressed as a value ranging from 0 to 1. The three detection layers in the Head correspond to three different sizes of feature maps obtained in the Neck. Within the feature maps, a predefined grid is established with three anchors of different aspect ratios. These anchors are used to store all the location and classification information based on the anchor prior frame in the channel dimension of the feature map for prediction and regression targets.

### CReToNeXt-YOLOv5 pig face expression recognition model

To effectively recognize facial expressions in pigs, the CReToNeXt-YOLOv5 model was utilized to classify and recognize four types of expressions in pig face images. A pig farm was set up in Shanxi Province, and video footage of pigs was collected using a video camera. Facial images of the pigs were then extracted from the videos. In order to improve the accuracy of expression recognition and achieve precise identification of targets, the EIOU loss function was employed instead of the CIOU loss function. Additionally, the Coordinate Attention mechanism was incorporated to enhance the model's sensitivity to expression features. The CReToNeXt module was also introduced to enhance the model's ability to detect subtle expressions.

**Figure 6:**
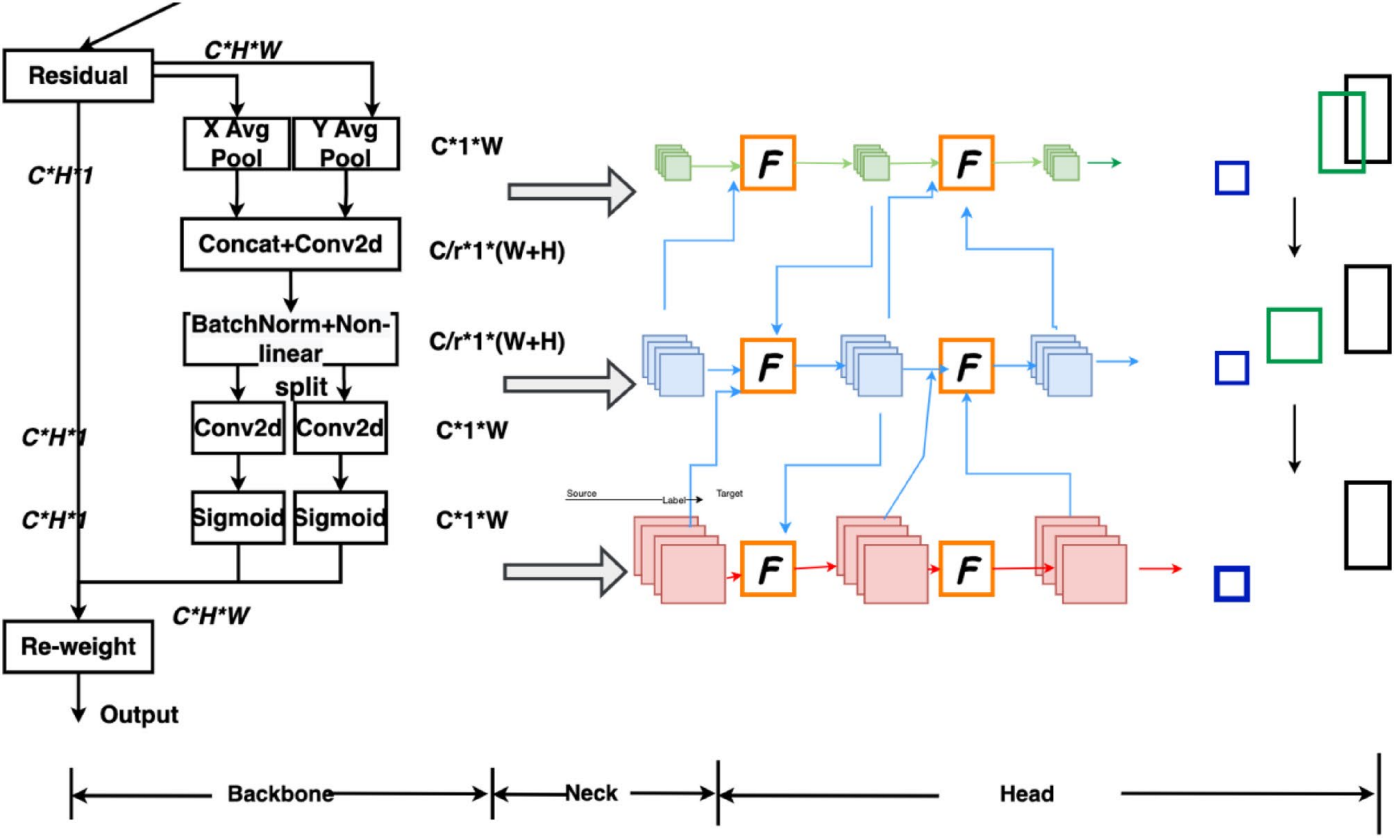
Improved framework for the CReToNeXt-YOLOv5 model: coordinate attention mechanism; EIOU loss function; CReToNeXt module.

The improvement points of the CReToNeXt-YOLOv5 model include incorporating the attention mechanism backbone, integrating the CReToNeXt module to enhance the model's neck and improve detection performance, and utilizing the optimized EIOU loss function in the Head section. The improvement framework of the CReToNeXt-YOLOv5 model is illustrated in Fig. 6.

#### Coordinate attention mechanism

**Figure 7:**
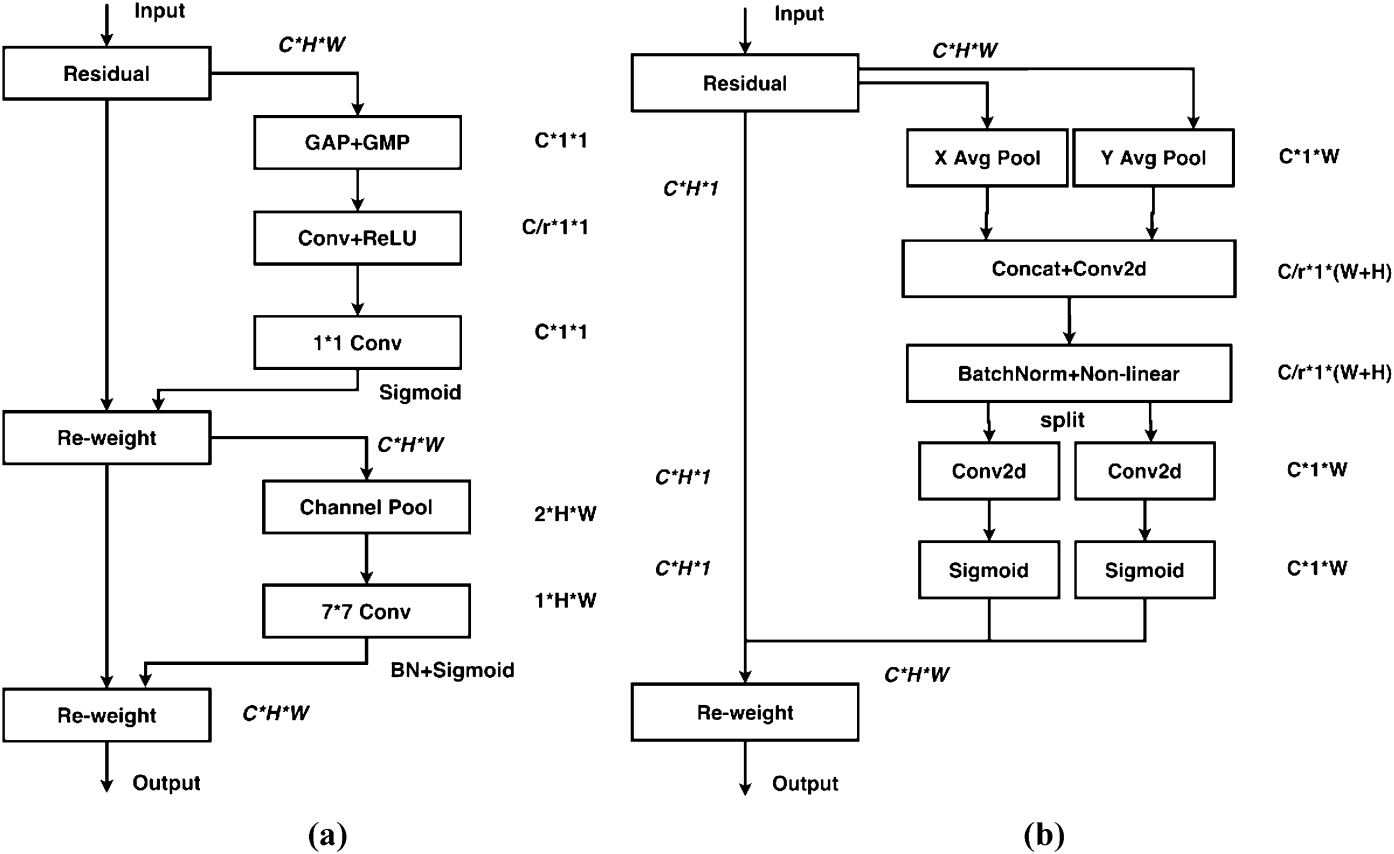
The structure of CBAM attentional mechanism compared with coordinate attention mechanism: (**a**) CBAM structure diagram; (**b**) coordinate attention structure diagram.

Attention mechanisms have been extensively studied to improve the capabilities of deep neural networks, particularly in capturing "what" and "where" information. However, their application in mobile networks significantly lags behind that of larger networks due to computational limitations. Squeeze-and-Excitation (SE) attention primarily focuses on encoding inter-channel information while disregarding positional information, which plays a crucial role in capturing object structures in visual tasks. The Convolutional Block Attention Module (CBAM) attempted to incorporate positional information by reducing the channel dimensionality of the input tensor and computing spatial attention using convolutions, as shown in Fig. 7a. However, visual tasks inherently involve long-range dependencies that cannot be effectively modeled solely through local relationships captured by convolutions.

The incorporation of the Coordinate Attention mechanism enhances the network's focus on the target to be detected and improves the detection results. When combined with the structure depicted in Fig. 7b below, the main concept of Coordinate Attention is as follows: the input features are separately pooled in the h-direction and w-direction, resulting in c × 1 × w and c × h × 1 feature maps. These pooled features are then concatenated. Since the dimensions are not the same, a direct concatenation would result in a broadcast mechanism, so they are adjusted to the same dimensions first. After the concatenation, a series of operations, such as a 1 × 1 convolution, is performed. At this stage, the number of convolution channels becomes 1/r of the original channels. The features are then split again and passed through sigmoid activation in different directions to obtain the attention coefficients, which are multiplied together.

##### Coordinate information embedding

The channel attention module globally encodes spatial information using the global pooling method. However, this approach leads to a loss of location information since the global spatial information is compressed in the channel descriptor during the encoding process. To address this issue, the global pooling is decomposed according to Eq. (2) and transformed into a one-dimensional feature encoding operation, preserving the location information.

2 $${{\text{Z}}}_{{\text{c}}}=\frac{1}{{\text{H}}\times {\text{W}}}{\sum }_{{\text{i}}=1}^{{\text{W}}}{\sum }_{{\text{j}}=1}^{{\text{W}}}{{\text{x}}}_{{\text{c}}}\left({\text{i}},{\text{j}}\right)$$

${\text{x}}$ is the input and each channel is first encoded along the horizontal and vertical coordinates using a pooling kernel of size (H,1) or (1,W) respectively. The output of the c-channel with height h can be expressed as:

3 $${{\text{Z}}}_{{\text{c}}}^{{\text{h}}}({\text{h}})=\frac{1}{{\text{w}}}\sum_{0\le {\text{i}}<{\text{W}}}{{\text{x}}}_{{\text{c}}}({\text{h}},{\text{i}})$$

Likewise, the output of the cth channel of width w is expressed as:

4 $${{\text{Z}}}_{{\text{c}}}^{{\text{w}}}({\text{w}})=\frac{1}{{\text{H}}}\sum_{0\le {\text{j}}<{\text{H}}}{{\text{x}}}_{{\text{c}}}({\text{j}},{\text{w}})$$

##### Coordinate attention generation

Coordinate Attention was developed based on three primary criteria: (a) simplicity in implementing the new transformation within the mobile environment. (b) The ability to accurately capture the region of interest by leveraging the captured location information. (c) Efficiently capturing relationships between channels. Once the new transformation in information embedding is performed, the previous transformation continues with a concatenate operation, followed by a convolutional transform function.

#### EIOU loss function

The EIOU loss function was adopted instead of the CIOU loss function used in the YOLOv5 algorithm to optimize the training model. This change significantly improves the accuracy of the pig face facial expression recognition algorithm and enables fast and accurate recognition of targets. The evolution of regression loss functions over the past few years is as follows: IOU_Loss → GIOU_Loss → DIOU_Loss → CIOU_Loss → EIOU_Loss. In this context, A represents the predicted bounding box, and B represents the ground truth bounding box. Based on this, the expression for the IOU_Loss can be written as:

5 $${\text{IOU}}\_{\text{Loss}}=1-{\text{IOU}}=1-\frac{{\text{A}}\cap {\text{B}}}{{\text{A}}\cup {\text{B}}}$$

When the predicted bounding box does not intersect with the ground truth bounding box, the IoU (Intersection over Union) does not reflect the actual distance between the two boxes, resulting in a IoU loss value of zero. This can negatively impact gradient backpropagation, making it difficult to train the model effectively. Furthermore, the IoU does not accurately represent the extent of overlap between the predicted and ground truth boxes. The DIoU (Distance-IoU) loss function addresses these issues by incorporating factors such as distance, overlap, and scale between the predicted and ground truth bounding boxes. This leads to more stable regression of the target bounding box. ${\text{b}}$ represents the centroid of the predicted box, ${{\text{b}}}^{{\text{gt}}}$ expresses the centroid of the real box,$\uprho$ is the Euclidean distance between the two centroids. ${\text{c}}$ denotes the diagonal distance between the prediction frame and the minimum closure region of the real frame. The DIOU_Loss expression is written as:

6 $${\text{DIOU}}\_{\text{Loss}}=1-{\text{DIOU}}=1-({\text{IOU}}-\frac{{\uprho }^{2}({\text{b}},{{\text{b}}}^{{\text{gt}}})}{{{\text{c}}}^{2}})$$

In YOLOv5s, the CIOU (Complete IoU) loss function was utilized to enhance the alignment between the predicted bounding box and the ground truth bounding box. It builds upon the DIOU (Distance-IoU) loss by incorporating additional penalties for the scale, length, and width of the detection bounding box. The expressions for the CIOU_Loss are as follows:

7 $${\text{CIOU}}\_{\text{Loss}}=1-{\text{CIOU}}=1-({\text{IOU}}-\frac{{\uprho }^{2}({\text{b}},{{\text{b}}}^{{\text{gt}}})}{{{\text{c}}}^{2}}-\mathrm{\alpha \gamma })$$

where $\mathrm{\alpha }$ denotes the weight parameter, which is expressed as:

8 $$\mathrm{\alpha }=\frac{\upgamma }{1-{\text{IOU}}+\upgamma }\upgamma$$

$\upgamma$ is adopted to measure the consistency of the aspect ratio, which is written as:

9 $$\upgamma =\frac{4}{{\uppi }^{2}}{({\text{arctan}}\frac{{\upomega }^{{\text{gt}}}}{{{\text{h}}}^{{\text{gt}}}}-{\text{arctan}}\frac{\upomega }{{\text{h}}})}^{2}$$

The aspect ratios, as relative values, introduce some ambiguity and do not consider the balance between difficult and easy samples (Zhang et al., 2022). In response to this, EIOU_Loss divides the aspect ratio into the difference in width and height between the predicted bounding box and the minimum external bounding box, based on the CIOU_Loss. The lengths and widths of the target and anchor boxes are then calculated separately. By minimizing the difference between the widths and heights of the target and anchor boxes, the convergence speed and regression accuracy are increased. Additionally, Focal Loss is introduced to balance the samples in the bounding box regression task. It is expressed as follows:

10 $${\text{EIOU}}\_{\text{Loss}}=1-{\text{EIOU}}=1-({\text{IOU}}-\frac{{\uprho }^{2}({\text{b}},{{\text{b}}}^{{\text{gt}}})}{{{\text{c}}}^{2}}-\frac{{\uprho }^{2}(\upomega ,{\upomega }^{{\text{gt}}})}{{{\text{c}}}_{\upomega }^{2}}-\frac{{\uprho }^{2}({\text{h}},{{\text{h}}}^{{\text{gt}}})}{{{\text{c}}}_{{\text{h}}}^{2}})$$

The advantages and disadvantages of each loss function were compared based on the three main geometric factors of bounding box regression (overlap area, centroid distance, and aspect ratio) (Table 2). When replacing the anchor frame loss function in YOLOv5s with EIOU_Loss, the performance showed significant improvement compared to the original IOU_Loss, DIOU_Loss, CIOU_Loss, and others.

**Table 2:** Comparison of loss functions.

Loss	Overlap	Centre point	Aspect Ratio	Advantages	Disadvantages
IOU_Loss	√	×	×	Scale invariance; non-negativity; homogeneity; symmetry; triangular inequality	If the two boxes do not intersect, it does not reflect the distance between the two boxes It does not accurately reflect the size of the overlap between the two boxes
GIOU_Loss	√	×	×	Solve the problem that loss equals 0 when there is no overlap between the detection box and the real box	GIOU degenerates to IOU when the detection frame and the real frame appear to be contained Slow convergence in the horizontal and vertical directions when the two frames intersect
DIOU_Loss	√	√	×	Convergence can be accelerated	The regression process takes into account the aspect ratio of the Bounding box and there is room for further improvement in accuracy
CIOU_Loss	√	√	√	The loss of detection frame scale, length and width has been increased so that the predicted frames will be more consistent with the real ones	Aspect ratios describe relative values and are subject to some ambiguity No consideration of the balance of difficult and easy samples
EIOU_Loss	√	√	√	The aspect ratio is replaced by a separate calculation of the difference in width and height, whereas Focal Loss is introduced to address the problem of unbalanced hard and easy samples	

#### CReToNeXt module

DAMO-YOLO, a forefront target detection approach, establishes a new benchmark by seamlessly integrating state-of-the-art technologies such as Neural Architecture Search (NAS), the innovative Reparameterized Generalized Feature Pyramid Network (RepGFPN), efficient lightweight heads, and strategic distillation augmentation. One of the cornerstone enhancements in DAMO-YOLO is the replacement of the CSPStage block found in the YOLOv5 architecture with the innovative CReToNeXt module, making the model more adaptive and proficient in object detection tasks, leading to unparalleled performance metrics^18^.

The CReToNeXt module, at its core, is designed as a convolutional neural network component, which specializes in the intricate task of image feature extraction. The design philosophy behind CReToNeXt is rooted in its bifurcated processing mechanism. Upon receiving an input, the module efficiently divides it into two distinct branches, each subjected to its specialized convolutional operations. These differentiated operations allow the module to capture a wider variety of image features, ensuring a comprehensive representation. Post these operations, the outputs from both branches are concatenated, preserving the richness of the extracted features.

Going deeper into its design intricacies, the CReToNeXt module can be expanded and made more robust. This is achieved by layering multiple instances of the BasicBlock_3 × 3_Reverse modules, each one building upon the previous, refining the extracted features further. To cater to varying scales and ensure that the module remains adaptive, an optional Spatial Pyramid Pooling (SPP) layer can be introduced, amplifying its feature extraction prowess across diverse image scales.

As the outputs from the two branches undergo the concatenation process, they are then channeled through a subsequent convolutional layer, which refines and outputs the final feature set. This intricate and detailed design of the CReToNeXt module not only augments YOLOv5's operational capacity but also refines its ability to capture and represent intricate semantic information contained within images, making it an invaluable tool in the world of object detection.

### YOLOv5 model evaluation indicators

To accurately judge the merits of the expression recognition models, Precision, mAP, Recall, and F1-score were selected for evaluation.

#### Precision

Precision indicates whether the detected area is always the correct area, with an ideal value of 1. The calculation formula is as in Eq. (11). ${\text{TP}}$: the true category of the sample is a positive example and the model predicts a positive outcome and correct prediction. ${\text{FP}}$: the true category of the sample is a negative example, but the model predicts it as a positive example and predicts it incorrectly.

11 $${\text{P}}=\frac{{\text{TP}}}{{\text{TP}}+{\text{FP}}}$$

#### mAP

mAP refers to the average accuracy and indicates the quality of the detection, with higher values representing better detection performance, as written in Eq. (12). ${\text{N}}$ denotes the total number of samples, ${\text{P}}({\text{n}})$ represents the size when n samples are identified at the same time,$\Delta {\text{P}}({\text{n}})$ represents the change in recall when the number of samples tested changes from n-1 to n,${\text{C}}$ expresses the number of categories in the multiclassification task.

12 $${{\text{P}}}_{{\text{mA}}}=\frac{{\sum }_{{\text{n}}=1}^{{\text{N}}}{\text{P}}({\text{n}})\Delta {\text{P}}({\text{n}})}{{\text{C}}}$$

#### Recall

The recall is calculated as Eq. (13), indicating whether the detection is comprehensive and whether all areas that should be detected are detected, with an ideal value of 1. ${\text{FN}}$: the true category of the sample is a positive example, whereas the model predicts it as a negative example and predicts it incorrectly.

13 $${\text{R}}=\frac{{\text{TP}}}{{\text{TP}}+{\text{FN}}}$$

#### F1-score

The F1-score refers to a measure of the classification problem. It is the summed average of precision and recall, with a maximum of 1 and a minimum of 0.

14 $${{\text{F}}}_{1}=\frac{2\times {\text{P}}\times {\text{R}}}{{\text{P}}+{\text{R}}}$$

## Results and discussion

In this segment, we embarked on rigorous experimentation with a meticulously curated dataset of swine facial expressions, the overarching aim being to discern the efficacy of our proposed CReToNeXt-YOLOv5 model. Our investigative trajectory bifurcated into two pivotal arms: an intrinsic validation of the model and a comparative analysis against contemporaneous target detection paradigms.

During the self-validation phase, we scrutinized the operational merits of coordinate attention mechanism, the EIOU loss function and the CReToNeXt module within our model. More specifically, under a harmonized experimental backdrop, we disabled these discrete components and juxtaposed results from their active and inactive states, offering insights into their individual and collective contributions to model performance. Further, to cast into sharp relief the competitive edge of our YOLOv5 paradigm in the domain of porcine facial expression recognition, we judiciously selected Faster R-CNN, YOLOv4, and YOLOv8 as comparative benchmarks.

It warrants emphasis that, juxtaposed against its subsequent iterations, YOLOv5 has garnered widespread vetting and endorsement, manifesting commendable maturity and stability. In our model selection crucible, beyond sheer performance, the stability and the collective acceptance within the broader research fraternity weighed heavily. While YOLOv7 dazzled with their precociously high mAPs during preliminary training, it simultaneously flagged potential overfitting susceptibilities, especially in context-specific datasets. This begets apprehensions regarding their robust applicability in real-world scenarios. Our research lens is keenly focused on the real-time recognition of porcine thermal stress expressions, making the resilience and reliability of the chosen paradigm paramount. Driven by these considerations, our pivot to the YOLOv5s as the foundational architecture was both deliberate and strategic, furnishing us with an expansive canvas for future optimizations and refinements.

### Performance of pig face expression recognition models in data training and validation

**Figure 8:**
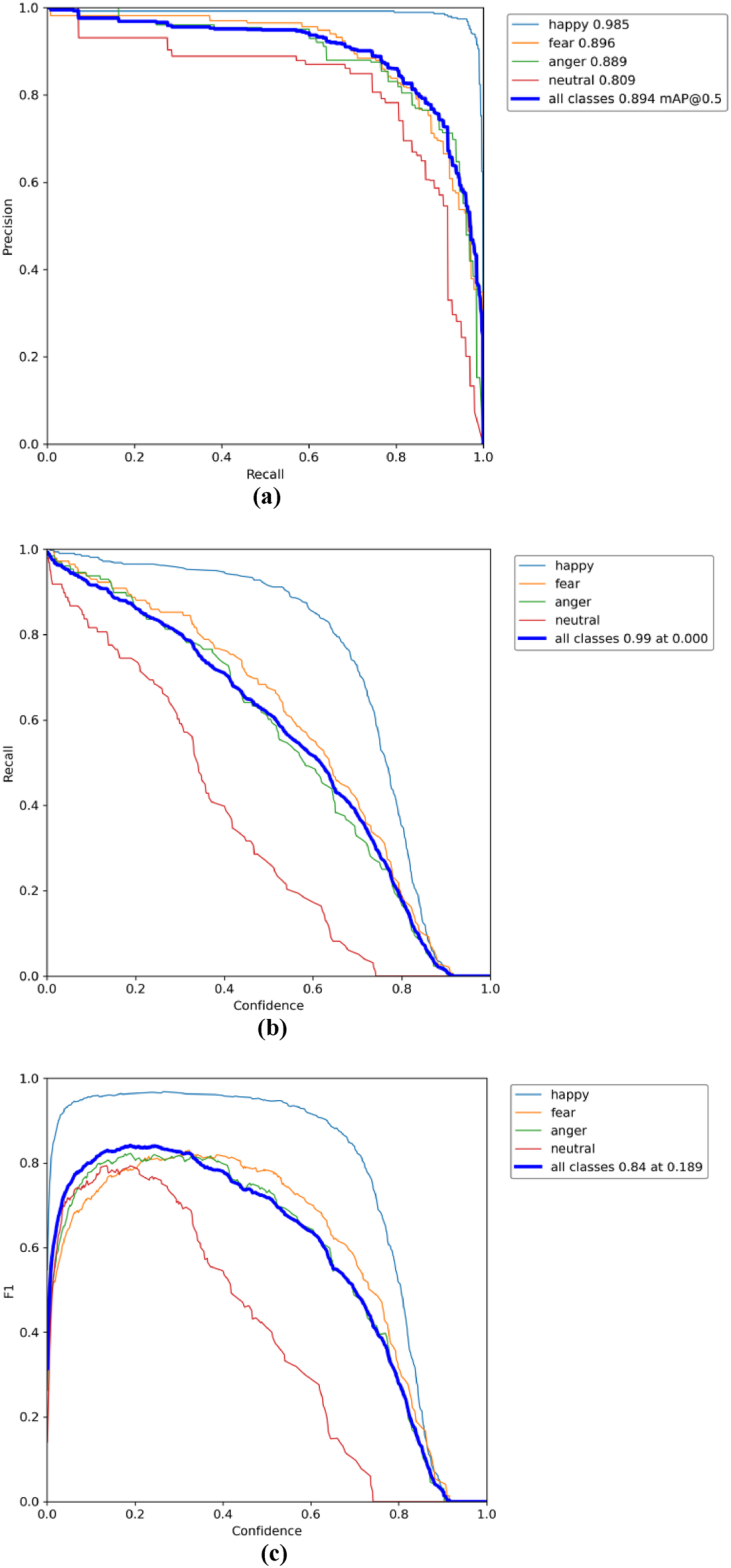
CReToNeXt-YOLOv5 model test results: (**a**) represents the precision–recall curve, showcasing an optimal trend of model performance over different thresholds. Solid blue line: represents the mean average precision (mAP) of the model, denoting an overall detection accuracy of 0.894 across all categories; (**b**) represents the recall rate, achieving a high mark of 0.99, indicating the model's proficiency in identifying relevant instances; (**c**) denotes the F1-score across all classes, peaking at 0.84 at a threshold of 0.189, highlighting the model's balanced performance between precision and recall.

As depicted in Fig. 8a, the Precision-Recall Curve offers a nuanced understanding of the CReToNeXt-YOLOv5 model's performance across different categories. One of the remarkable takeaways from this representation is the model's confidence scores. Generally, a higher confidence score is synonymous with superior detection ability, and our model exemplifies this trend, suggesting its proficient discernment in object detection tasks. The mAP of 0.894 further consolidates this, emphasizing the model's robustness and high overall detection accuracy. Transitioning to the Recall Curve, illustrated in Fig. 8b, the value of 0.990 denotes the model's adeptness in target detection, although there might still be minor opportunities for refinement in ensuring every genuine instance is identified. Perhaps most tellingly, the F1-score, showcased in Fig. 8c, encapsulates a holistic performance metric, harmonizing precision and recall. An F1-score is an indispensable indicator, often signifying the balance a model maintains between false positives and false negatives. In our context, an F1-score of 0.84 is not only commendable but indicative of a well-optimized balance the CReToNeXt-YOLOv5 model has achieved between precision and recall, setting a benchmark for subsequent model iterations and research endeavors.

**Figure 9:**
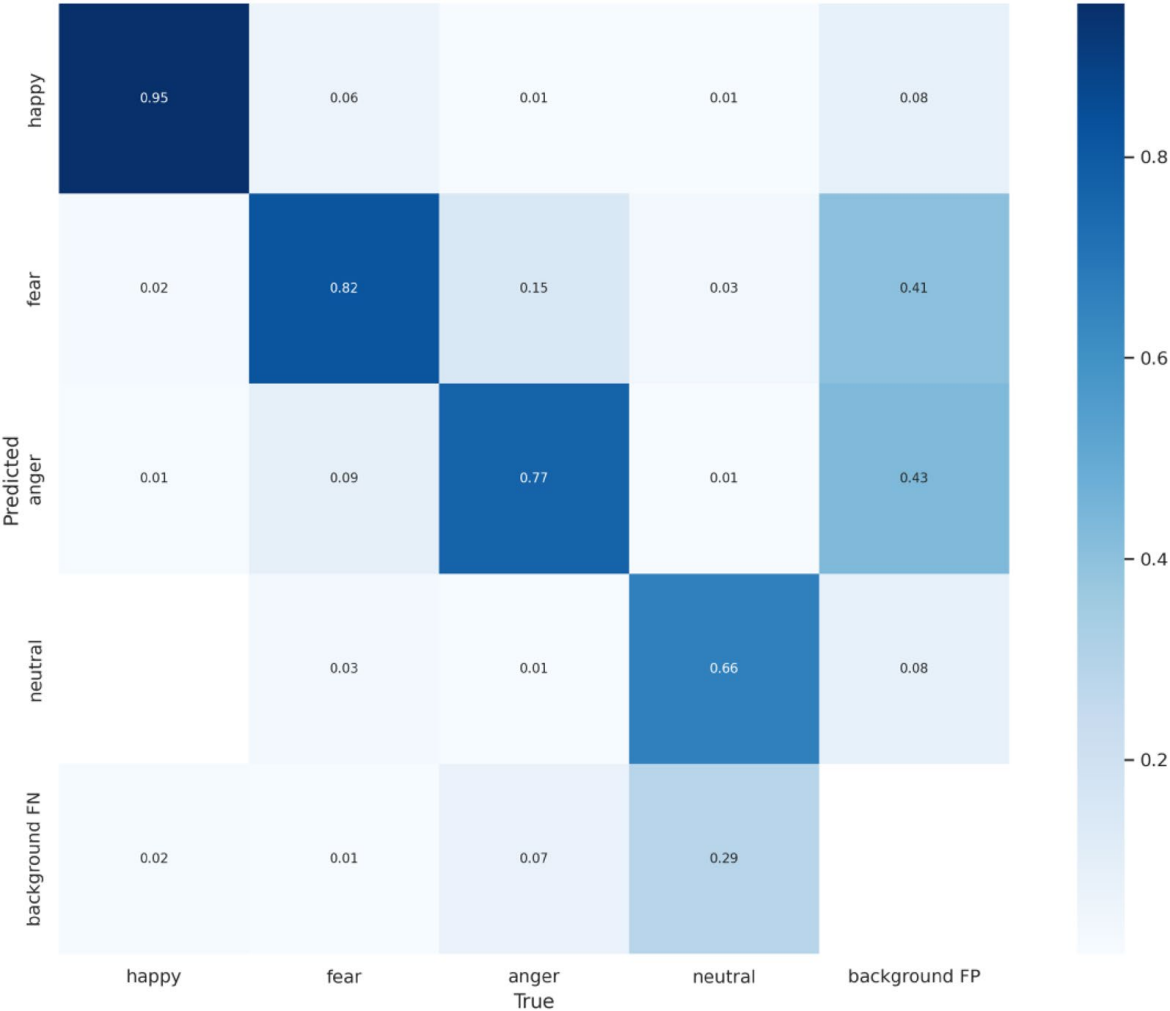
Confusion Matrix for the CReToNeXt-YOLOv5 Model.

The confusion matrix, as illustrated in Fig. 9, offers a holistic understanding of the CReToNeXt-YOLOv5 model's performance in discerning distinct pig facial expressions. Impressively, the model shows a particularly strong aptitude in identifying the "happy" expression, registering it with a high accuracy. However, certain challenges emerge when it comes to distinguishing between the nuanced shades of "fear" and "anger". The model occasionally confuses "fear" for "happy" and vice-versa, indicating a potential area for improvement in distinguishing between positive and more subdued or negative expressions.

Furthermore, a notable aspect arises in the identification of the "neutral" expression. This category, inherently subtle by nature, poses a challenge, with a significant percentage being overlooked and classified as background. This might point towards the model's sensitivity to subtle cues or the need for richer training data for this particular class. Another interesting observation is the occasional misclassification of what is essentially background noise as distinct emotional states. This points towards the potential for refining the model's noise filtering capabilities.

In summary, while the model showcases robustness in certain emotional categories, there's room for refinement. The confusion matrix serves as a guide, highlighting areas like distinguishing subtleties in certain emotions and improving background noise filtering, thus paving the way for more nuanced and precise future iterations.

**Figure 10:**
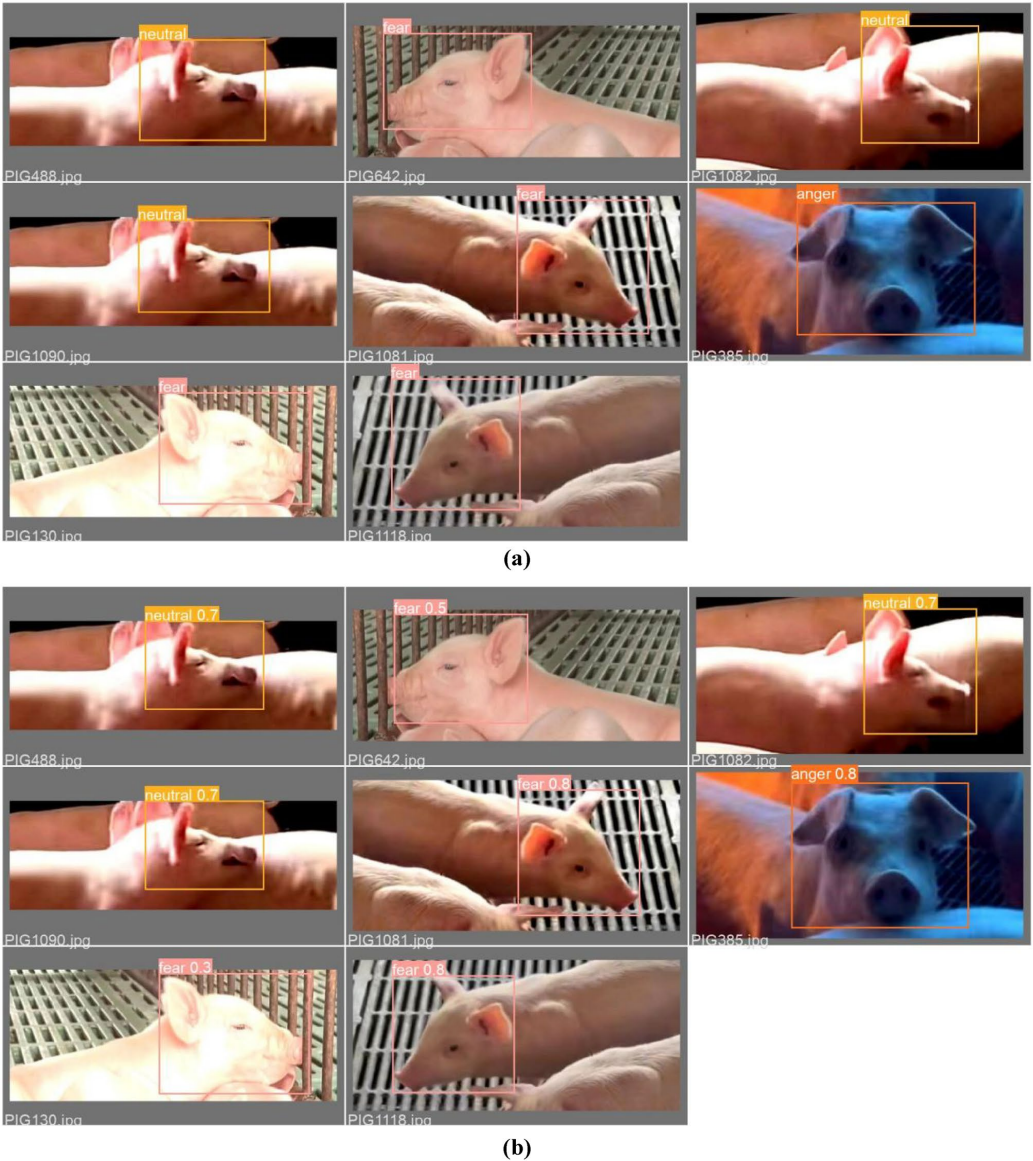
CReToNeXt-YOLOv5 model test results: (**a**) Val_batch_labels; (**b**) Val_batch_pred.

Figure 10 elucidates the test outcomes of the CReToNeXt-YOLOv5 model, juxtaposing validation batch labels (depicted in part (a)) against their corresponding predictions (highlighted in part (b)). At a glance, one can discern the model's nuanced proficiency in capturing the spectrum of pig facial expressions, with a commendable degree of accuracy in identifying emotions such as 'neutral', 'fear', 'anger', and particularly 'happy'. Delving deeper into the insights offered by this visual representation, the model's prowess is particularly underscored in its ability to accurately decode 'happy' expressions, reflecting a high degree of precision in the range of 80% to 90%. This not only underscores its finesse in distinguishing overtly positive states but also sets a benchmark for its robustness in this specific domain.

However, the waters become slightly murkier when navigating the realms of more subdued or complex emotions like 'fear' and 'anger', both registering at a 50% accuracy level. These figures, while respectable, suggest a room for refinement, potentially indicating the challenges in differentiating these intricate emotional states, especially given their close physiological and visual similarities in certain contexts. The 'neutral' category, historically a challenge due to its inherent subtlety, boasts a detection accuracy of 70%. This highlights the model's capability in discerning subtle cues, an attribute that's paramount when deciphering states that lack pronounced emotional undertones.

In essence, Fig. 10 presents a mosaic of the CReToNeXt-YOLOv5 model's strengths and potential avenues for enhancement. While it excels in detecting unequivocal emotions like happiness, there lies an exciting frontier in refining its sensitivity to the more intricate tapestry of emotions, ultimately aiming for an iteration that encapsulates the full breadth and depth of pig facial expressions with unparalleled precision.

### Self-validation results and analysis of the CReToNeXt-YOLOv5 model

**Figure 11:**
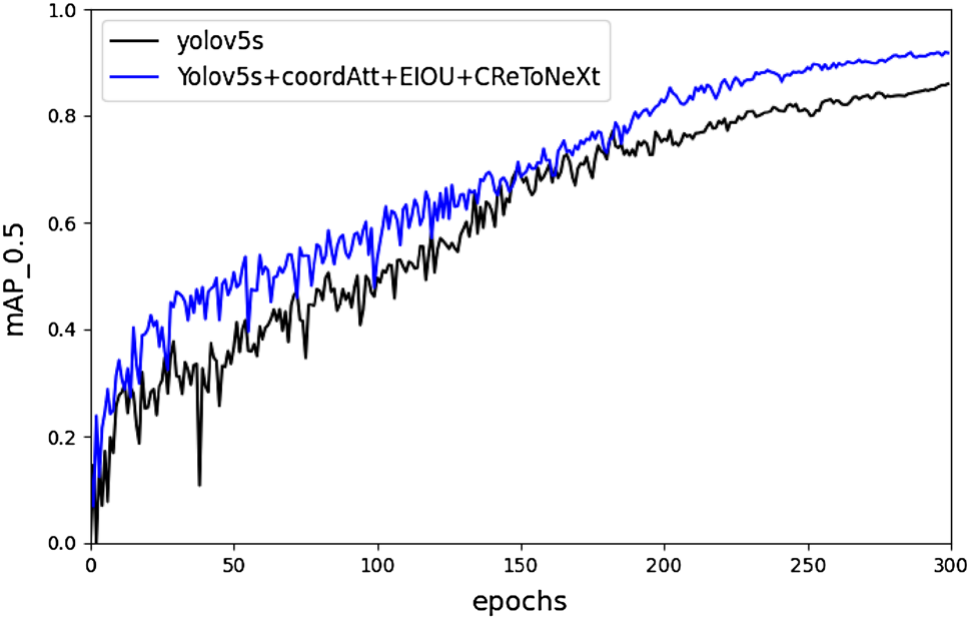
The mAP comparison graph between the CReToNeXt-YOLOv5 model and the YOLOv5 model.

The experimental results on the self-labeled pig expression image dataset are shown in Table 3. The mAP of the CReToNeXt-YOLOv5 model was 89.4%, which is a significant improvement of 6.7% compared to the original YOLOv5 model. The CReToNeXt-YOLOv5 model showed improvements in various categories, such as a 5.4% improvement in angry expression recognition, 9% improvement in fear, 1.1% improvement in happy, and 11.8% improvement in neutral. Figure 11 visually demonstrates the improved accuracy of pig face expression detection achieved by the CReToNeXt-YOLOv5 model.

**Table 3:** Self-validation results of the CReToNeXt-YOLOv5 model.

Model	Precision				mAP	Recall	F1-score
	Happy	Neutral	Fear	Anger			
Yolov5s	0.974	0.691	0.806	0.835	0.827	0.779	0.77
Yolov5s + EIOU	0.978	0.689	0.839	0.824	0.832	0.798	0.77
Yolov5s + CBAM	0.981	0.760	0.858	0.841	0.860	0.815	0.65
Yolov5s + coordAtt	0.984	0.803	0.864	0.831	0.870	0.827	0.67
Yolov5s + coordAtt + EIOU	0.984	0.775	0.868	0.877	0.876	0.826	0.82
Yolov5s + coordAtt + EIOU + CReToNeXt	0.985	0.809	0.896	0.889	0.894	0.990	0.84

In the course of refining the model for improved accuracy and robustness, two potent attention mechanisms, CBAM and Coordinate Attention Mechanism, were integrated into the Yolov5s framework, and their individual contributions were meticulously evaluated. A thorough analysis reveals that the integration of the Coordinate Attention Mechanism, as evidenced by its metrics, offered a more superior performance enhancement compared to the CBAM mechanism. Specifically, when observing the mAP, which is a crucial indicator of a model's precision and recall balance, the Coordinate Attention Mechanism-equipped model slightly outperformed its CBAM counterpart, reflecting a marginally better overall object detection capability. Furthermore, the model enhanced with the Coordinate Attention Mechanism also showcased a marked improvement in detecting the "Neutral" emotion, an inherently challenging task given the subtlety of neutral expressions. This suggests that this mechanism might offer the model an edge in discerning subtle features in the dataset. While both mechanisms provided commendable results, the decision to favor the Coordinate Attention Mechanism over CBAM was driven by its overall superior and consistent performance across various metrics. The choice underscores the importance of iterative experimentation and data-driven decisions in model optimization, ensuring that the chosen refinement offers both theoretical and practical improvements to the system's capabilities.

**Figure 12:**
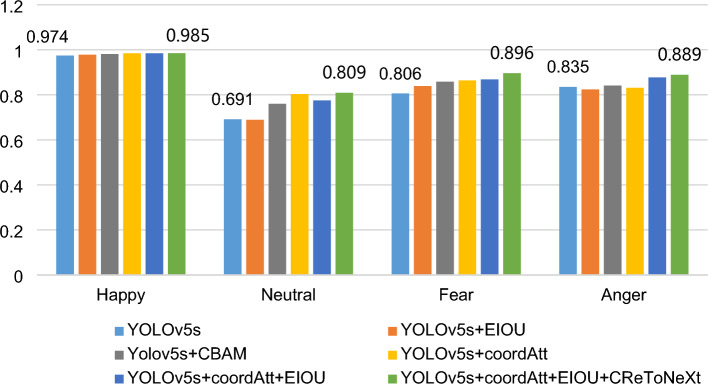
Based on the improved YOLOV5 model, the comparison of the detection accuracy of four expression categories.

Figure 12 provides a comparison of the prediction results for the four expression categories among the optimized models. It is evident that the Happy category is easily distinguishable, with an accuracy exceeding 97% for all optimized models. On the other hand, the Neutral category proves challenging to identify accurately. The Happy category dataset was specifically chosen to be in a controlled pig feeding environment with fewer variables, making it easier to distinguish. In contrast, the Neutral category dataset encompasses a diverse environment with numerous factors, leading to potential prediction errors.

The inclusion of the Coordinate Attention mechanism addresses this issue by decomposing the channel attention into two separate 1-dimensional feature encoding processes. These processes aggregate features along two spatial directions individually. This approach allows for capturing remote dependencies along one spatial direction while preserving precise location information along the other. The resulting feature maps are encoded as direction and location-aware attention maps, which can be applied in a complementary manner to the input feature maps. This enhances the representation of objects of interest and effectively improves the accuracy of Neutral category recognition.

Incorporating the Coordinate Attention Mechanism into the YOLOv5s framework for pig facial expression detection epitomizes the blend of precision and spatial understanding in object detection techniques. For a nuanced task like detecting varying pig facial expressions, the minute, spatially-specific details play a pivotal role. Traditional models often struggle with capturing these subtle cues, especially when expressions share commonalities. With the Coordinate Attention Mechanism, the model is endowed with the ability to selectively focus on regions of the feature map that are most informative for a particular expression. By weighting these regions more heavily in the subsequent layers, the model becomes more adept at distinguishing between expressions that might otherwise appear similar.

The integration of the EIOU loss function into the YOLOv5s framework for pig facial expression detection epitomizes the evolution of object detection methodologies. The EIOU (enhanced intersection over union) loss function builds on the foundational IOU loss, renowned for its efficacy in bounding box regression tasks. However, EIOU further refines this by addressing certain pitfalls inherent in traditional IOU computations.

For the nuanced task of detecting varied pig facial expressions, the precision of bounding boxes becomes paramount. Misalignments or inaccuracies in these bounding boxes can drastically affect model performance, as the differences between certain expressions can be subtle and localized. The EIOU loss function specifically mitigates such issues by considering not only the overlap between predicted and actual bounding boxes but also the geometric and positional characteristics of these boxes. By doing so, it ensures a more holistic matching, capturing finer discrepancies that might be overlooked by traditional loss functions. Furthermore, EIOU minimizes the penalization of predictions that are close yet not perfectly aligned, making the learning process more forgiving and stable. This nuanced approach to bounding box regression, facilitated by the EIOU loss function, makes it a crucial component in enhancing the YOLOv5s model's accuracy for pig facial expression detection. The sophisticated error backpropagation driven by EIOU ensures that the model's predictions are consistently refined, leading to superior detection results across varied scenarios.

Incorporating the CReToNeXt module into the YOLOv5s framework for detecting the four facial expressions of pigs represents a significant advancement in object detection models. The integration capitalizes on the strengths of the CReToNeXt module, leveraging its superior feature extraction and harmonization capabilities.

At the heart of the CReToNeXt module lies its ability to bifurcate input data into two distinct pathways, subjecting each to different convolutional operations. This dual-path mechanism is instrumental in capturing both fine-grained, localized features and broader, contextual details from the input image. For the task at hand, discerning nuanced facial expressions in pigs, the importance of this approach cannot be overstated. Subtle variations in facial muscle movement, which often distinguish one emotion from another, are meticulously captured and emphasized through this method. Another compelling advantage of CReToNeXt's incorporation is its flexibility in feature map dimensionality. Given the inherent disparities in facial expressions, ensuring that the model can accommodate a granular control over feature map channels is critical. This is especially pertinent for complex emotions that might manifest through a combination of minute facial cues. Furthermore, the optional inclusion of the Spatial Pyramid Pooling layer in the CReToNeXt module facilitates multi-scale feature extraction. Given the variance in size and orientation that facial expressions can have, depending on the pig's posture and distance from the camera, this multi-scale approach ensures that the model remains robust and invariant to such changes.

In summary, the integration of the CReToNeXt module into the YOLOv5s architecture provides a holistic improvement, particularly for the challenging task of pig facial expression recognition. By adeptly managing multi-scale feature extraction, capturing fine-grained facial nuances, and harmonizing broader context, CReToNeXt sets the stage for unparalleled accuracy and precision in this domain.

### Comparison of target detection models of the same type

For the intricate task of detecting facial expressions in pigs, the foundational algorithm of choice becomes pivotal. While the progression in the YOLO series is evident through its advancements in versions like YOLOv7, their lightning-fast convergence to 99% accuracy within the initial 20 training rounds sounds promising but rings alarm bells. Such rapid accuracy spikes, especially on specific datasets, often hint at a lurking risk of overfitting. Overfit models, despite their resounding performance during training, can struggle profoundly when introduced to novel real-world data. Their high sensitivity to training data intricacies often leaves them bewildered when confronted with the multifarious and unpredictable nuances of real-world scenarios.

Our primary focus lies not just in achieving high numbers during training but ensuring steadfast and reliable performance in genuine applications. This is especially true for the challenging arena of recognizing thermal stress-induced expressions in pigs, a scenario filled with subtle facial cues and varying environmental conditions. Therefore, a reliable and robust algorithm is more valuable than a hypersensitive one, leading us to base our studies on the YOLOv5s architecture. YOLOv5s offers a commendable balance of accuracy and speed without prematurely plateauing, granting us ample room for tailored enhancements and adjustments.

But how does YOLOv5s fare against other contemporaries in its league? When juxtaposed against the likes of Faster R-CNN, YOLOv4, and YOLOv8, YOLOv5s's prowess becomes evident. Faster R-CNN, a two-stage detector, employs a Region Proposal Network (RPN) to earmark potential object regions, which are then refined and classified. While this methodological depth often equips Faster R-CNN with remarkable precision, especially with smaller and densely packed objects, it comes at the cost of increased computational load, potentially making real-time applications challenging. YOLOv4, sharing YOLO's hallmark single-stage detection architecture, exhibits a balanced performance, boasting improvements over its predecessors but not quite matching the finesse of YOLOv5. By segmenting the entire image into grid cells and performing simultaneous object detection and classification, it offers real-time speeds, making it particularly attractive for applications demanding prompt feedback. YOLOv8 stands as a state-of-the-art (SOTA) model, building upon the successful foundation of its predecessors in the YOLO series. Introducing novel features and improvements, YOLOv8 aims to enhance performance and flexibility. Noteworthy innovations include a redesigned backbone network, a novel Anchor-Free detection head, and a new loss function.

**Figure 13:**
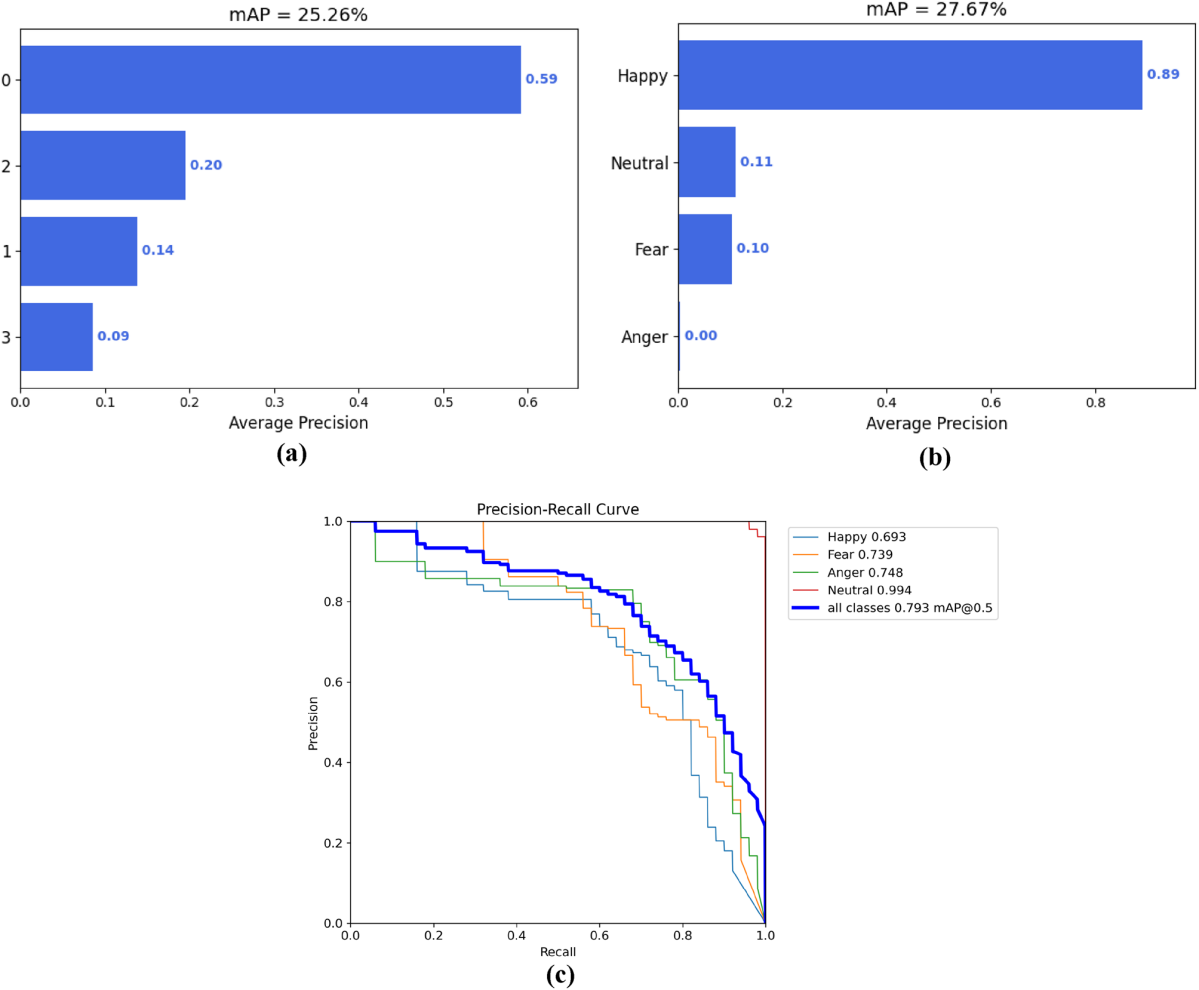
Comparative analysis of mAP for expression recognition: (**a**) faster R-CNN model, (**b**) YOLOv4 model, and (**c**) YOLOv8 model.

In Fig. 13a, the Faster R-CNN's Mean Average Precision (mAP) stood at 25.26%, with "happy" expressions being detected at a promising 59%. However, its ability to detect "neutral", "fear", and especially "anger" expressions lagged significantly. In contrast, Fig. 13b shows YOLOv4's results with an mAP of 27.67%. Interestingly, while its accuracy for detecting "happy" expressions soared to an impressive 89%, it struggled profoundly with "anger" expressions, failing to detect them entirely. As depicted in Fig. 13c, the YOLOv8 model excels in recognizing the "happy" facial expression, underscoring its outstanding performance in identifying joyful facial cues. However, the confidence levels for "fear" (0.759) and "anger" (0.709) are relatively lower, indicating a potential need for further optimization to enhance the model's sensitivity to these specific expressions. Meanwhile, the performance in detecting the "neutral" expression is the least satisfactory (0.495). This disparity may reflect challenges that the model encounters when dealing with certain facial expressions, warranting closer attention and targeted improvements.

In conclusion, while YOLOv7 might seem tantalizing due to their blazing-fast convergence, the shadows of overfitting they cast render them less reliable for real-world applications like pig facial expression recognition. On the other hand, YOLOv5, when weighed against Faster R-CNN, YOLOv4, and YOLOv8, emerges as an algorithm that not only provides rapid detection but also allows considerable enhancement leeway, setting the stage for precision-tuned, application-specific advancements.

### Analyzing the proficiency of CReToNeXt-YOLOv5 across varied emotional categories

The efficacy of the CReToNeXt-YOLOv5 model in discerning diverse facial expressions in pigs is noteworthy, and to truly appreciate its prowess, one must delve deeper into its performance across specific emotional states. When considering the angry expressions in pigs, characterized by features like deepened wrinkles and wide-open eyes, the CReToNeXt-YOLOv5 stands out. Its dual-path mechanism, integral to its architecture, adeptly captures both the pronounced features and the more subtle nuances, such as the upward pull of the upper lip.

Fear, another intricate emotion in pigs, reveals itself through cues like slightly closed eyes, reduced wrinkles, and droopy ears. The model excels here too, due in large part to the Spatial Pyramid Pooling layer within the CReToNeXt module, which facilitates multi-scale feature extraction. This dynamic feature ensures that the model retains its accuracy in detecting fear regardless of variables like the pig's posture or its distance from the camera.

The manifestations of happiness in pigs, though appearing clear-cut with slightly closed eyes, an upward tugging of the upper lip, and ears pulled back, present their own challenges. These expressions, especially evident in an eating state, require a model that can precisely pinpoint such fleeting moments. The model's attention mechanism, which permits selective focus on crucial regions like the mouth and eyes, rises to this challenge, ensuring that these moments of joy are not overlooked.

Perhaps the most challenging to detect are the neutral expressions, primarily because of their lack of distinctive features. Yet, it is this very state of normalcy that offers crucial baseline data on a pig's emotional health. The model's Coordinate Attention Mechanism is particularly crucial here, granting the model the finesse to detect even the minutest deviations from a neutral state, a feat that many traditional models might falter at.

Furthermore, the CReToNeXt-YOLOv5 model's proficiency is not merely a product of its individual components but rather their synergistic operation. The dual-path design of the CReToNeXt, combined with the spatial specificity of the CoordAtt mechanism and the holistic perspective provided by the EIOU function, collectively contribute to its impeccable accuracy. This robust amalgamation ensures that the model doesn't just detect but also precisely classifies each facial expression, setting a new benchmark in the realm of facial expression recognition in livestock.

### Limitations in Swine expression analysis with CReToNeXt-YOLOv5

In delving into our CReToNeXt-YOLOv5 model aimed at recognizing and classifying swine facial expressions, we've discerned certain nuanced intricacies that can be viewed as potential limitations to our study. Primarily, our dataset predominantly captures swine behavior in specific contexts, particularly under the "Happy" category, where most data are accrued during feeding times. This specificity could inadvertently narrow the model's generalizability when presented with joyful pig expressions outside of feeding scenarios, despite offering a clear benchmark for our analyses. Additionally, the diversity inherent in the "Neutral" category, encompassing myriad environments, introduces variability which, in turn, could challenge the model's predictive precision. Practical challenges also arose during data acquisition, given the dynamics of swine movement, camera angles, and varying lighting conditions, all of which could subtly skew the quality of data collected. While we've endeavored to mitigate these by employing a multi-camera and multi-angle strategy, the inherent unpredictability of these variables could still influence the model's holistic efficacy. Furthermore, there's a plausible risk that our dataset might not fully represent the gamut of swine breeds, age groups, or health conditions, potentially introducing an undercurrent of bias. As we venture forward, it's imperative for subsequent research to both acknowledge and address these subtleties, striving for a more encompassing tool for swine facial expression recognition.

## Conclusions

In our quest to comprehend the intricate web of emotions exhibited by livestock, we recognized that facial expressions serve as a pivotal channel. These expressions not only hint at the emotional well-being but also shed light on the psychological health of the animals, making their accurate detection and classification of paramount importance. Addressing inherent challenges, such as the pigs' rudimentary facial muscle structure and nuances in expressions that often evade detection, we embarked on creating a versatile multi-scene pig facial expression dataset. The richness of this dataset, encompassing varied survival scenarios, emotional states, and diverse perspectives, forms a comprehensive foundation for pig facial expression recognition. Our pioneering work led to the development of the CReToNeXt-YOLOv5 model, tailored to adeptly classify and recognize pig facial expressions. The remarkable strides we made in boosting the expression recognition rates can be attributed to strategic integrations like the CoordAtt attention mechanism, the innovative EIOU loss function, and the essence of the CReToNeXt module. This synergy doesn't merely bolster the technical prowess of machine vision but resonates deeply with the overarching goal of advancing humane and intelligent livestock farming practices.

Benchmarked against stalwarts in the realm of target detection, CReToNeXt-YOLOv5's superiority emerges undeniably. While the YOLOv5 model, known for its rapid inference and accuracy, sets a commendable standard, our model not only matches but surpasses it, registering an impressive mAP of 89.4% across four expression categories. This is a tangible improvement of 6.7% against YOLOv5 and even more pronounced when juxtaposed with models like Faster R-CNN and YOLOv4, outperforming them by 64.14% and 61.73%, respectively. Furthermore, with the inclusion of the YOLOv8 model in our comparative analysis, it exhibits a mean average precision (mAP) of 79.3%. Noteworthy is the significant 10.1% superiority demonstrated by our model over YOLOv8. This amalgamation of findings not only emphasizes the sustained outperformance against established models but also accentuates the substantial lead maintained by our model, thereby reaffirming its prowess in terms of both accuracy and inference speed.

Nevertheless, our model, despite its accomplishments, isn't without its challenges. The diverse environments where the Neutral category database is located, compounded with multifaceted influencing factors, have sometimes yielded suboptimal recognition rates. Addressing these intricacies, refining our approach, and expanding our dataset with varied scenes and pig breeds stand as our future endeavors. By achieving these, we aim to bolster the model's robustness and ensure its broader applicability, not just in research settings but in real-world livestock management scenarios, ultimately uplifting animal welfare standards.

## Data Availability

All code generated during this study are available in the Github repository. Links to the code are provided in the below hyperlinked text. Code of CReToNeXt-YOLOv5 project: https://github.com/Nielili1998/pig-face-expression-recognition. The datasets generated and/or analysed in the current study are not publicly available due to data being collected in collaboration with third-party pig farms, but are available from the respective authors upon reasonable request. Confirmation that these pigs were not handled by the authors during the study. Confirmation that all experimental protocols have been approved by the designated institution and/or licensing board. Confirm that all methods were performed in accordance with relevant guidelines and regulations.
